# Star Identification Based on Multilayer Voting Algorithm for Star Sensors

**DOI:** 10.3390/s21093084

**Published:** 2021-04-28

**Authors:** Meiying Liu, Xin Wei, Desheng Wen, Hu Wang

**Affiliations:** 1 Xi’an Institute of Optics and Precision Mechanics of Chinese Academy of Sciences, Xi’an 710119, China; liumeiying@opt.ac.cn (M.L.); ven@opt.ac.cn (D.W.); wanghu@opt.ac.cn (H.W.); 2 University of Chinese Academy of Sciences, Beijing 100049, China

**Keywords:** star identification, triangle unit voting, singular values feature, star sensor

## Abstract

This paper describes the multilayer voting algorithm, a novel autonomous star identification method for spacecraft attitude determination. The proposed algorithm includes two processes: an initial match process and a verification process. In the initial match process, a triangle voting scheme is used to acquire candidates of the detected stars, in which the triangle unit is adopted as the basic voting unit. During the identification process, feature extraction is implemented, and each triangle unit is described by its singular values. Then the singular values are used to search for candidates of the imaged triangle units, which further improve the efficiency and robustness of the algorithm. After the initial match step, a verification method is applied to eliminate incorrect candidates from the initial results and then outputting the final match results of the imaged stars. Experiments show that our algorithm has more robustness to position noise, magnitude noise, and false stars than the other three algorithms, the identification speed of our algorithm is largely faster than the geometric voting algorithm and optimized grid algorithm. However, it takes more memory, and SVD also seems faster.

## 1. Introduction

Independent and reliable attitude measurement plays an important role in modern space missions. Star sensors supply the more precise attitude reference data than other attitude determination devices, such as the sun sensor, magnetometer, and gyroscope [1,2,3]. Therefore, star sensors have become popular in attitude determination for interplanetary spacecraft and orbiting satellites in the last two decades [4].

Star identification is a key technique for star sensors to execute their attitude determination functions. Generally, two typical operating modes are included in the identification process: lost-in-space (LIS) mode and tracking mode. When a star sensor is activated for the first time or misses the attitude data, it will operate in LIS mode. In this case, there is not any prior attitude information, and a full-sky autonomous star identification algorithm must be performed to acquire an initial attitude. Once having the established initial attitude, the star sensor turns to tracking mode and then performs a recursive method [5] utilizing the prior attitude information to search for the corresponding matches between imaged stars and guide stars in a very small sky region. Thus, the star identification in tracking mode is much easy than it in LIS mode.

In the last forty years, a number of full-sky autonomous star identification methods have been proposed to solve the lost-in-space problem. These algorithms are roughly partitioned into three categories [6]: pattern recognition algorithms, artificial intelligence algorithms and subgraph isomorphism algorithms.

The basic idea of the pattern recognition approach is to associate each star with an individual signature or pattern vector determined by surrounding stars, and then the match is achieved by finding the most similar star pattern from the onboard database. The grid algorithm by Padgett and Kreutz-Delgado [7], the typical pattern-based star identification method, and the performance of this method is good in terms of efficiency, memory size and identification rate. Unfortunately, the probability of correctly choosing the closest neighboring star is low, and it significantly limits its performance, particularly when there is noise in the captured star image. Na et al. [8], Clouse and Padgett [9], Lee and Bang [10], and Aghaei and Moghaddam [11] proposed some modifications to improve the grid method, but it is still an issue. To overcome the drawback of selecting a subaltern star in the grid algorithm, Silani and Lovera [12] subsequently proposed the polestar algorithm based on a radial pattern. However, the pattern in the polestar method only utilizes the distribution information in radial orientation and cannot describe the distribution of the neighboring stars comprehensively. Thus it performs poorly in the occasion with large star position noise [13]. Zhang et al. [14] proposed a novel algorithm with radial pattern and cyclic pattern features by adding cyclic information in generating patterns, which reduces the ambiguous matches in the initial match step. Nevertheless, the cyclic pattern is not reliable due to sensitivity to noise. In 2007, the log-polar transform (LPT) was introduced into the star identification methods by Wei [15], namely the LPT star identification algorithm. This method is robust to noise, but comparing pattern feature vectors in the LPT algorithm is extremely time-consuming. A star identification based on “flower code” was proposed by Gong [16] in 2009. It is robust for false stars but also less efficient due to its complex pattern match step.

The artificial intelligence algorithms mainly utilize neural networking or bionic intelligence to identify imaged stars captured by the star sensor. For example, the methods based on adaptive ant colony algorithm [16], multilayer SOM neural network [17], representation learning algorithm [18], or convolutional neural networks (CNN) [19] have been designed to solve the star identification problem. These algorithms simplify the matching process and have lower memory demands. However, their high complexity, considerable training time and the need for a massively parallel architecture limit these approaches in engineering projects.

The subgraph–isomorphism-based algorithms treat star identification as an example of subgraph isomorphism, which considers stars sharing the same field of view (FOV) as vertices in a graph and their angular separations as corresponding edges, respectively. Identification is acquired when the graph constructed by detected stars in FOV is identified uniquely with a part of the database. These methods utilize star pairs, triples, pyramids or polygons to construct the onboard database, such as the triangle algorithm [20,21,22], pyramid algorithm [23], and geometric voting algorithm [24]. In 1995, Liebe [2] proposed the pioneering triangle algorithm where angular distances of triples are used as features for identification, but redundant matches often occur with position noise, and a large memory resource is also required. Based on the original triangle algorithm, some modifications were proposed by scholars to refine the triangle construction scheme, accelerate the triangle match process or promote the reliability of the verification step. However, redundant matches or incorrect matches are still issues because of the low feature dimension of the triangle. Later Mortari et al. [23] innovated the pyramid algorithm, in which four stars are employed to form the geometric polygon, and the k-vector approach is adopted in the searching procedure. This method shows robustness to false stars, but its runtime turns unacceptable with the number of false stars increasing. A geometric voting technique was proposed by Kolomenkin et al. [24], it finds the corresponding relationship between detected stars and catalog stars by voting with many angular distances, and the time required for star identification is low. However, when spikes present in the star image, the performance of the geometric voting algorithm severely degrades.

Some scholars have proposed other approaches in recent years, such as image correlation matching, KMP, color ratios, Hausdorff distance, hidden Markov models, and hash maps. In 2003, a new identification algorithm was proposed in [25] utilizing the singular value decomposition (SVD) to simplify the star identification. In the algorithm, the singular value is robust to position noise, while the process of ranking detected stars brightness is sensitive to the magnitude noise, which results in identification failures.

To refine the efficiency and robustness of the star identification, we present a multilayer voting star identification algorithm in this paper. Our algorithm utilizes the triangle unit as the basic element in the first voting process to get candidates for some detected brighter stars, and then the verification method based on angular distance voting is performed to determine the final matching results. Compared with the grid, geometric voting and SVD algorithms, the performance of our method is much better on the occasion of star position noise and star magnitude noise. In addition, it also achieves a higher identification rate when the false stars present in the image. The contributions of our study are listed as follows: (1). For the star identification algorithms adopting a voting scheme, the triangle unit is selected as a voting unit for the first time; (2). The voting scheme in our algorithm utilizes two different voting units in the initial voting process and the verification voting process, respectively, to improve the ability of the algorithm; (3). The identification rate of our algorithm is much higher with noise, and it is also efficient.

This paper is organized as follows: Section 2 describes our algorithm in detail, including the initial match and the verification. Simulations are performed to evaluate the identification rate and efficiency of the proposed method and compare its performance with the other three algorithms In Section 3. In Section 4, several conclusions are summarized and outlined.

## 2. Algorithm Description

In this section, the implementation details about the multilayer voting algorithm are provided. The triangle unit construction and feature values extraction are first presented. Then the onboard database build and its corresponding structure are described thoroughly. Finally, the main algorithm steps, along with its pseudocode and an identification example, are respectively presented in detail.

### 2.1. Triangle Unit and Feature Extraction

**Figure 1:**
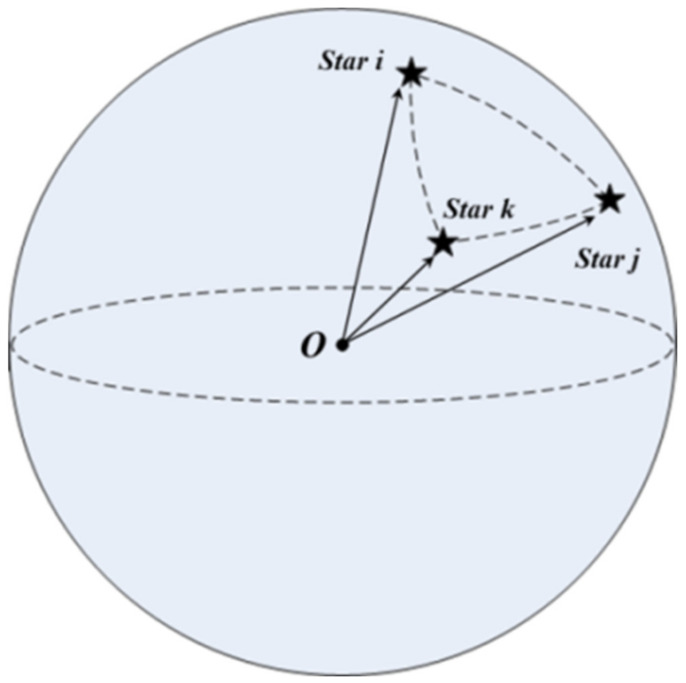
Illustration of the triangle unit.

The proposed algorithm constructs the triangle units in the field of view (FOV) as the basic vote unit and then implements the triangle voting process by matching the extracted features. Hence, the triangle unit and feature extraction are briefly introduced as follows:

For any three stars *i*, *j*, *k* in the celestial sphere, they can form the triangle unit $T_{i,j,k}$ with their corresponding direction vectors in the celestial coordinate system $p_{i}^{c}$, $\;p_{j}^{c}$, and $p_{k}^{c}$, as shown in Figure 1, and its unit matrix $M_{C}$ is given by:

(1) $$M_{c}=\left[\begin{array}{ccc} p_{i}^{c} & p_{j}^{c} & p_{k}^{c} \end{array}\right]$$

where

(2) $${p_{t}^{c}=[\cos(\alpha_{t})\cos(\delta_{t})\;\sin(\alpha_{t})\cos(\delta_{t})\;\sin(\delta_{t})]}^{T}$$

where $\alpha_{t}$ is the ascension, $\delta_{t}$ is the declination of the star *t* (*t* = *i*, *j*, *k*).

After determining the unit matrix $M_{C}$, the SVD transformation is utilized to extract the feature values:

(3) $$M_{c}=U\sum V^{T}$$

where *U* and *V* are both orthogonal matrices, ∑ is a diagonal matrix with three singular values $v_{1}$,$\;v_{2}\;$ and $v_{3}$, which includes features of the unit matrix $M_{C}$.

The invariance of singular values with coordinate transformation was first proved by Juang in 2003. Then Zhu et al. verified its robustness under position noise. After implementing SVD transformation, three singular values in ∑ are sorted in descending order, and the elements $v_{1}$ and $v_{2}$ maintain the primary geometric feature information of the unit $T_{i,j,k}$, thus they are selected as feature values for our algorithm. By utilizing the SVD transformation, the triangle unit is transformed to $v_{1}$ and $v_{2}$. To improve the searching efficiency of the method, the feature values $v_{1}$ and $v_{2}$ are then discretized. As Equation (4) shows, the discretized singular value ${\hat{v}}_{1}\;$ is obtained from the singular value $v_{1}$ divided by $\Delta v_{1}$, and the discretized singular value ${\hat{v}}_{2}\;$ is obtained from the singular value $v_{2}$ divided by $\Delta v_{2}$. The discretized feature values ${\hat{v}}_{1}$ and ${\hat{v}}_{2}$ will be used as the match features to obtain candidates in the initial match process:

(4) $${\hat{v}}_{1}=ceil(v_{1}/\Delta v_{1})\cdot {\hat{v}}_{2}=ceil(v_{2}/\Delta v_{2})$$

where *ceil*(*x*) rounds *x* to the nearest integer no less than *x*, $\Delta v_{1}\;$ and $\Delta v_{2}\;$ are the discretized unit for the singular value $v_{1}\;$ and $v_{2}$, respectively. For example, if $v_{1}$ is 1.7081, $\Delta v_{1}$ is $5.0\times 10^{-5}$, then ceil (1.7081/$5.0\times 10^{-5}$) value is 34,162, so the discretized feature values ${\hat{v}}_{1}$ is 34,162. In the same way, when $v_{2}$ is 0.1272, $\Delta v_{2}$ is $5.0\times 10^{-4}$, the discretized feature values ${\hat{v}}_{2}$ is 254.

### 2.2. Onboard Database Generation

**Figure 2:**
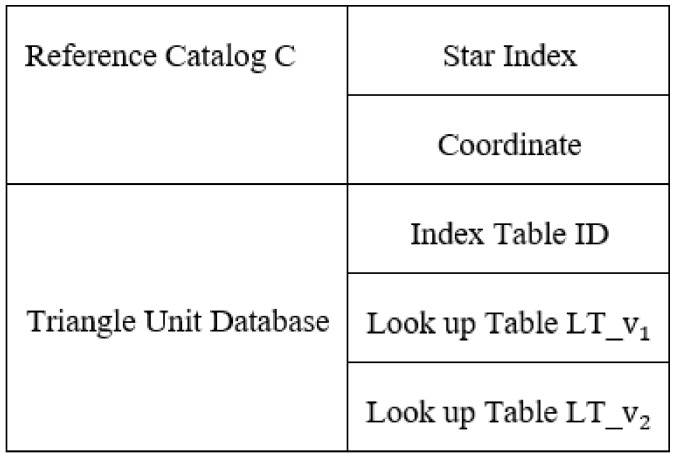
Structure of onboard database.

The onboard database of the multilayer voting method includes a Reference catalog ***C*** and triangle unit database, which consists of Index table ***ID*** and two features Look up tables (namely, LT_$v_{1}$ and LT_$v_{2}$). The detailed database structure is given in Figure 2, and its memory size is about 2.1 MB.

Assuming a FOV of 25 × 25 ° and stars with apparent magnitudes down to 5.5 Mv can be detected by the star sensor, and 2859 stars brighter than 5.5 Mv in the SAO J2000 catalog are selected. After discarding the variable stars and binary stars, the Reference catalog ***C*** and Index table ***ID*** are first constructed for the algorithm. Considering the number of the triangle units built by all catalog stars will be too huge, and it results in high computational complexity and massive memory requirement, so only part of the triangle units is selected and stored in the ***ID***. The generation procedure of the Reference catalog ***C*** and Index table ***ID*** is as following steps:

**Step1**: the 100,000 points are distributed evenly on the sphere by the method in [26], then a star image is generated for each point as the boresight;

**Step2**: in each star image, select the brightest N catalog stars to generate $C_{N}^{3}$ triangle units by these stars;

**Step3**: store the index, ascension and declination of the selected stars in catalog ***C*** and the indexes of three stars in each triangle unit in the index table ***ID***, respectively. The discretized singular values lay the foundation of the two feature lookup tables. The feature value ${\hat{v}}_{1}\;$ Loo kup table LT_$v_{1}$ and the feature value ${\hat{v}}_{2}$ Look up table LT_$v_{2}$ is built based on the discretized singular values ${\hat{v}}_{1}\;$ and ${\hat{v}}_{2}$., respectively. The details are described as follows: Each triangle unit in the Index table ***ID*** is dealt with Equations (1)–(4) to extract its corresponding feature values ${\hat{v}}_{1}\;$ and ${\hat{v}}_{2}$. When ${\hat{v}}_{1}\;$ of the triangle unit in the *i*-th row of Index table ***ID*** is *m*, then enter the index *i* into the *m*-th row of the LT_$v_{1}$. When ${\hat{v}}_{2}.\;$ of the triangle unit in the *i*-th row of the Index table ***ID*** is *n*, then enter the index *i* into the *n*-th row of the LT_$v_{2}$. When all triangle units in Index table ***ID*** are processed by the above procedure, then LT_$v_{1}$ and LT_$v_{2}$ are completed.

### 2.3. Multilayer Voting Algorithm

The proposed method starts with inputting the coordinates and brightness of the detected stars in the captured image. Then the algorithm acquires candidates for several detected bright stars by voting for the triangle units formed by these bright stars. The catalog star with the maximum votes is considered as a candidate of the corresponding sensor star. The resulting identities are correct for the most part, but some are erroneous. Hence, a filter step must be added to eliminate incorrect match results. The multilayer voting algorithm involves two voting processes: the initial match step based on the triangle unit voting and the verification step based on star pairs voting. The former includes the feature values extraction and determining candidates from the voting results; the latter utilizes angular distances among the detected bright stars to find the largest cluster group from candidates and then acquire final pairings between sensor stars and catalog stars.

#### 2.3.1. Initial Match

The brightest N stars are selected as the sensor reference stars (SRSs), then generate $C_{N}^{3}$ triangle units by these stars, forming the set {$T_{i,j,k}^{s}$}, where $T_{i,j,k}^{s}$ is the triangle unit constructed by stars *i*, *j*, *k* (∈[1,N]) in the image, which are denoted by $s_{i}$, $s_{j}$, $s_{k}$.

For each triangle unit, the feature values are extracted by the SVD transformation described in Section 2.1, in which the unit matrix $M_{b}$ of the triangle unit $T_{i,j,k}^{s}$ can be formed by three direction vectors in the star sensor body system $p_{i}^{b}$,$\;p_{j}^{b}$, and $p_{k}^{b}$, it can be obtained using Equations (5) and (6):

(5) $$M_{b}=\left[\begin{array}{ccc} p_{i}^{b} & p_{j}^{b} & p_{k}^{b} \end{array}\right]$$

where

(6) $$p_{t}^{b}=\frac{1}{\sqrt{x_{t}{}^{2}+y_{t}{}^{2}+f^{2}}}{\left[\begin{array}{ccc} x_{t} & y_{t} & f \end{array}\right]}^{T}\cdot (t=i,\;j,\;k)$$

where ($x_{t}$,$\;y_{t}$) is the image coordinates of the detected star $s_{t}$, *f* is the optical length, and ||·|| denotes Euclidean norm operation.

After discretizing the singular values, the LT_$v_{1}$ and LT_$v_{2}$ are searched according to the discretized feature values ${\hat{v}}_{1}^{s}$ and ${\hat{v}}_{2}^{s}$ obtained from the imaged triangle unit $T_{i,j,k}^{s}$. For all triangle units’ feature values ${\hat{v}}_{1}^{c}$ and ${\hat{v}}_{2}^{c}$ in the database that meet the feature values ${\hat{v}}_{1}^{s}$ and ${\hat{v}}_{2}^{s}$ simultaneously (Equation (7)) will be considered as the candidates of the imaged triangle unit $T_{i,j,k}^{s}$, and then they form a vote set K:

(7) $$\left|{\hat{v}}_{1}^{s}-{\hat{v}}_{1}^{c}\right|\leq Th_{1}\;\left|{\hat{v}}_{2}^{s}-{\hat{v}}_{2}^{c}\right|\leq Th_{2}$$

where $Th_{1}$ and $Th_{2}$ are the tolerance thresholds in the two feature values match process.

**Figure 3:**
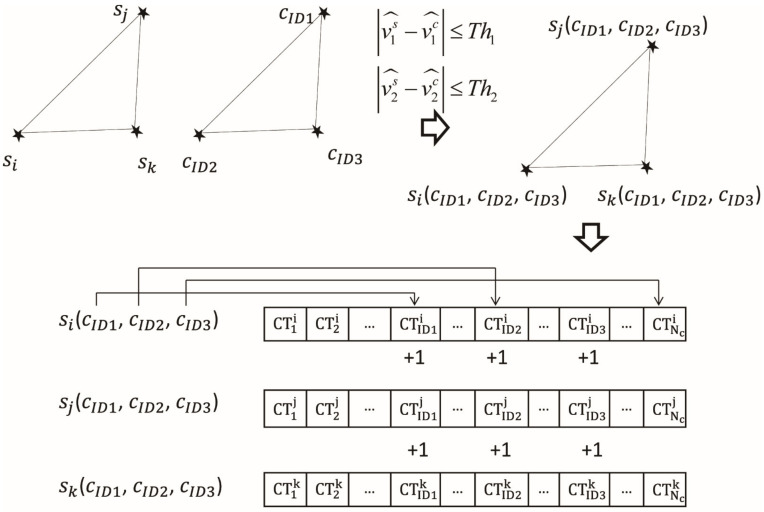
Vote process in the initial match step.

Then for each element *T* in *K*, a vote is cast for the identity of the three corresponding detected stars *i*, *j*, *k* in the captured image. For example, if a catalog triangle unit $T_{\mathrm{ID}1,\mathrm{ID}2,\mathrm{ID}3}^{c}\in K$, where $T_{\mathrm{ID}1,\mathrm{ID}2,\mathrm{ID}3}^{c}$ represents the triangle unit constructed by stars indexed as ID1, ID2, ID3 in the star catalog C, which are denoted by $c_{\mathrm{ID}1}$, $c_{\mathrm{ID}2}$, $c_{\mathrm{ID}3}$, then all image stars $s_{i}$, $s_{j}$*,* $s_{k}$ will get votes from catalog stars $c_{\mathrm{ID}1}$, $c_{\mathrm{ID}2}$, $c_{\mathrm{ID}3}$ as possible identities for them. For the sensor star $s_{i}$, its counters (${\mathrm{CT}}_{1}^{i}$,${\mathrm{CT}}_{2}^{i}$…${\mathrm{CT}}_{N_{c}}^{i}$) of catalog stars that correspond with $c_{\mathrm{ID}1}$, $c_{\mathrm{ID}2}$, $c_{\mathrm{ID}3}$ add one, $N_{c}$ is the guide star number in catalog C, the same operation is done for counters belong to other sensor stars $s_{j}$ and $\;s_{k}$, respectively, as shown in Figure 3.

Once all $C_{N}^{3}$ triangle units in the image are processed by the above-mentioned procedure, and the triangle voting step has ended. Then the values of counters associated with each sensor reference star are compared, and the catalog star with the maximum votes is considered as the candidate for the sensor reference star.

#### 2.3.2. Verification

Once the initial match step is over, the candidates of SRSs are determined. However, there may be incorrect matches in the resulting initial identities, and it will cause erroneous attitude estimation, which is fatal for space missions. Therefore, a verification method based on star pairs voting is implemented to exclude these spurious candidates and obtain final matching results in our method.

During the verification process, we assign a counter for each candidate star and set the counters’ value to zero at the beginning of the voting. The angular distance between sensor stars $S_{i}$ and $S_{j}\;$ is $\theta_{ij}^{s}$, and the angular distance between their corresponding candidates $C_{i}$,$\;C_{j}$ is $\theta_{ij}^{C}$. The detected star pair ($S_{i}$*,*$\;S_{j}$) is considered a match with the star pair ($C_{i}$*,*$\;C_{j}$) if the difference between $\theta_{ij}^{s}$ and $\theta_{ij}^{C}$ is less than the tolerance threshold ε, as described in Equation (8):

(8) $$\left|\theta_{ij}^{s}-\theta_{ij}^{c}\right|\leq \varepsilon$$

If Equation (8) is satisfied, $S_{i}\;$ gets a vote from the candidate $C_{i}$, $S_{j}\;$ gets a vote from the candidate $C_{j}$, then the counters associated with candidate stars $C_{i}\;$ and $C_{j}$ increase by one. Repeat it until the last one detected star pair. The stars with correct candidates will support each other, while stars with incorrect candidates get few votes. The largest cluster of candidates is found by the star pairs voting method in the verification step, and if the number of elements included in the cluster is larger than the threshold ver_min, then our algorithm reports a success and outputs final identification results. Otherwise, it returns the failure.

### 2.4. Pseudocode Description

The key parameters used in the multilayer voting method are listed in Table 1, and they are determined as follows:

(1) N is the maximum number of selected stars in an image to construct triangle units. Larger N means more true triangle units will be involved in the initial matching process, which contributes to identifying star images with false stars and magnitude noise but also require more memory and time consumption. In this paper, we set N = 10, generating 199,562 triangle units;
(2) ver_min is the threshold for the largest cluster group size, which determines the error identification rate of our method. According to the analytic model in [27], ver_min is set to 3, considering the false attitude is unacceptable;
(3) $\Delta v_{1}\;$ and $\Delta v_{2}\;$ are the minimum discretized unit for the singular value $v_{1}\;$ and $v_{2}$, respectively. Obviously, these parameters affect the robustness of the algorithm to position noise. Considering the identification rate and time performance, we choose $\Delta v_{1}$ = 0.00005 and $\Delta v_{2}$ = 0.0005, more details about the parameters selection are described in Section 3.1.

All functions in the proposed method are listed in Table 2, and a high-level pseudocode description of the multilayer voting method is presented in Algorithm 1. The algorithm input is the coordinates and brightness of the N brightest detected stars in the image. Once the triangle voting procedure is completed, all candidates can be brought into the verification (can) to search for the largest cluster group among them and then the identified stars indexes for sensor reference stars returned.**Algorithm 1** The pseudocode of the proposed algorithm**Begin** counter = 0*counter **for** *i* = 1 to *i* = N − 2 **do** **for** *j* = *i* + 1 to *j* = N − 1 **do** **for** *k* = *j* + 1 to *k* = N **do**    [${\hat{v}}_{1}$,$\;{\hat{v}}_{2}$] = *feature_value* (*i, j, k*)    [*unit_num*, *K*] = *search_match_unit* (${\hat{v}}_{1}$,$\;{\hat{v}}_{2}$, LT_$v_{1}$, LT_$v_{2}$)      **for** *p* = *1* to *p* = *unit_num* **do**
       [*id1*, *id2*, *id3*] = *unit_starid* (*K*, *p*)           *counter*[*i*] = *add_counter* (*i, id1, id2, id3*)           *counter*[*j*] = *add_counter* (*j, id1, id2, id3*)           *counter*[*k*] = *add_counter* (*k, id1, id2, id3*)     **end for** **end for** **end for** **end for** **for**
*q* = 1 to *q* = N **do** *can*[*q*] = *find_candidate* (*counter[q]*) **end for** *recog_flag* = *verification* (*can*) **if** *recog_flag* = 0 **return** *NULL* **else return** *id* **End**

### 2.5. An Identification Example

**Figure 4:**
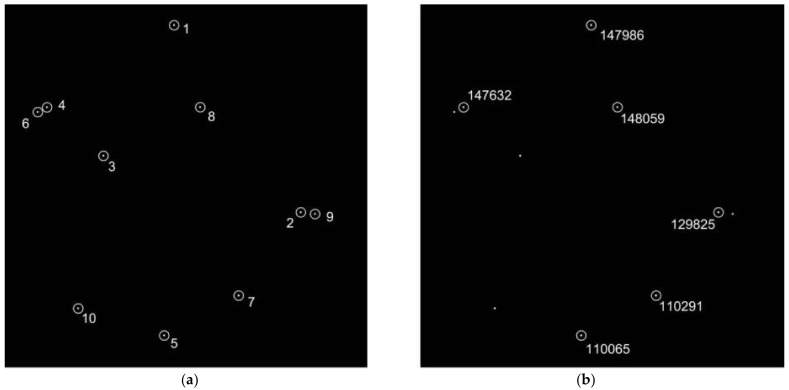
An identification example: (**a**) star image to be identified; (**b**) identification results with the proposed method.

An identification example is given to illustrate how the multilayer voting algorithm works. A star image is simulated randomly in Figure 4a. As listed in Table 3, the coordinates of 10 selected SRSs are inputted into the algorithm. The position noise is set to 1.5 pixels, and brightness noise is set to 0.5Mv, resulting in two extra stars (image star 3 and 6), which are not included in the catalog, are picked as sensor reference stars. Moreover, one false star (image star 9) is also added at random locations. The discretized unit $\Delta v_{1}\;$ and $\Delta v_{2}\;$ is $5.0\times 10^{-5}$ and $5.0\times 10^{-4},$ respectively, and the *ver_min* is 3.

After implementing the initial match step, each sensor reference star obtains its candidate. As shown in Table 3, the candidate for image star 10 is not the correct match. Moreover, there are also candidates for two extra stars (image star 3 and 6) and one false star (image star 9). To acquire the correctly identified results, the verification step is performed. During this step, mismatches are not included in the largest cluster group and removed from the final matching results of the algorithm. Figure 4b shows the final identification results of Figure 4a.

## 3. Implements and Results

Simulations and real star image tests were done to evaluate the performance of the proposed multilayer voting algorithm under different noise levels. In this paper, successful identification of a star image needs to meet two requirements: (1) There should be neither true stars identified incorrectly nor false stars identified. (2) no less than three true stars are identified correctly. Moreover, we adopted the geometric voting [24], optimized grid [11] and SVD [25] algorithms as references, since they are the most representative algorithms and exhibit excellent performance in space missions, and then compared the capabilities of the proposed with these reference methods. All four-star identification methods are programmed with MATLAB and performed on a Core 3.4 GHz PC with Microsoft Windows environment. In simulations, the star sensor used a 25 × 25° FOV, the image plane was made up of 1024 × 1024 pixels, and its minimum sensitivity was 5.5 Mv. The SAO J2000 star catalog, including 9040 stars with a magnitude of no more than 6.5 Mv, was utilized for generating simulated star images.

### 3.1. Parameter Selection

The parameters $\Delta v_{1}\;$ and $\Delta v_{2}$ used in discretizing the singular values $v_{1}$, $v_{2}\;$ are important to correctly identify sensor stars. Considering the identification rate, the discretized units should set at a big value, especially when sensor stars are of large position error. However, more redundant candidates of the voting unit may emerge, requiring more run time to implement the triangle voting process. Nevertheless, if $\Delta v_{1}\;$ and $\Delta v_{2}$ is set at small values, there will be riskier to exclude correct candidates.

**Figure 5:**
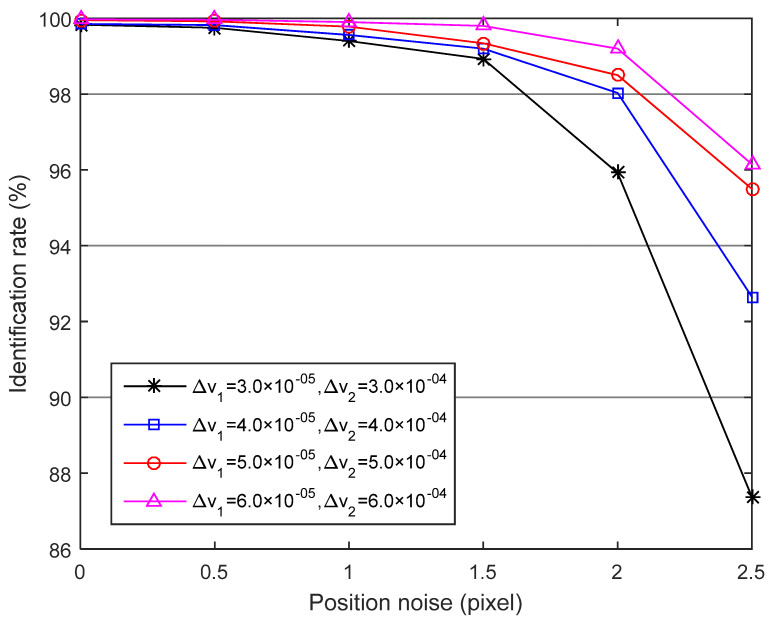
Identification rate versus positional noise with the different discretized units.

Figure 5 shows the relationship between the identification rate of the multilayer voting method and $\Delta v_{1}$,$\;\Delta v_{2}$ as the standard deviation of position, noise increases from 0 to 2.5 pixels, while the magnitude noise is fixed at 0.3 Mv. We can see that the algorithm performance degrades quickly when the centroid position errors increase more than 1.5 pixels with $\Delta v_{1}$,$\;\Delta v_{2}$ is $3.0\times 10^{-5}$,$\;3.0\times 10^{-4}$.Note that the algorithm achieves an identification rate at a higher level in two cases where $\Delta v_{1}$,$\;\Delta v_{2}$ is set to $5.0\times 10^{-5}$,$\;5.0\times 10^{-4}$ and $6.0\times 10^{-5}$,$\;6.0\times 10^{-4}$. Furthermore, when $\Delta v_{1}$,$\;\Delta v_{2}$ is $5.0\times 10^{-5}$,$\;5.0\times 10^{-4}$, the corresponding run time is 35.3 ms, while the time increases to 43.9 ms for $\Delta v_{1}$,$\;\Delta v_{2}$ is $6.0\times 10^{-5}$,$\;6.0\times 10^{-4}$. In this experiment, we choose $\Delta v_{1}$ = $5.0\times 10^{-5}$, $\Delta v_{2}$ = $5.0\times 10^{-4}$ by considering both robustness and time performance.

### 3.2. Comparison and Analysis

In this section, the influence of three sources of noise, namely, star position noise, star magnitude noise, and false stars, on identification rates of the proposed method, geometric voting method, the optimized grid method, and the SVD method are mainly discussed. In the simulations, the key parameters of three comparison algorithms are set as follows: for the geometric voting method, the parameter the distance threshold σ = 0.06°; for the optimized grid method, the parameter resolution g = 80 and match threshold min_mat = 4; for the SVD method, the parameter threshold ε = 0.01.

The position noise added to the detected stars leads the projected centroids in the sensor plane of these stars to deviate from their theoretical locations. In addition, we also add the random Gaussian noise to the star brightness to simulate star magnitude noise, which results in some brighter stars whose magnitude falls below the instrumental threshold to be lost and stars dimmer than the sensor threshold to actually appear in our simulated star images. Apart from these measurement noises, there may also be false stars in the captured image because of planets, observed spacecraft, radiation, space fragments and hot spots. In experiments, the parameters in the algorithms were fixed, one noise source varied linearly, while the others were fixed at a typical level, 10,000 tests were conducted to evaluate the identification rates of four different methods for each noise condition using the Monte Carlo method.

**Figure 6:**
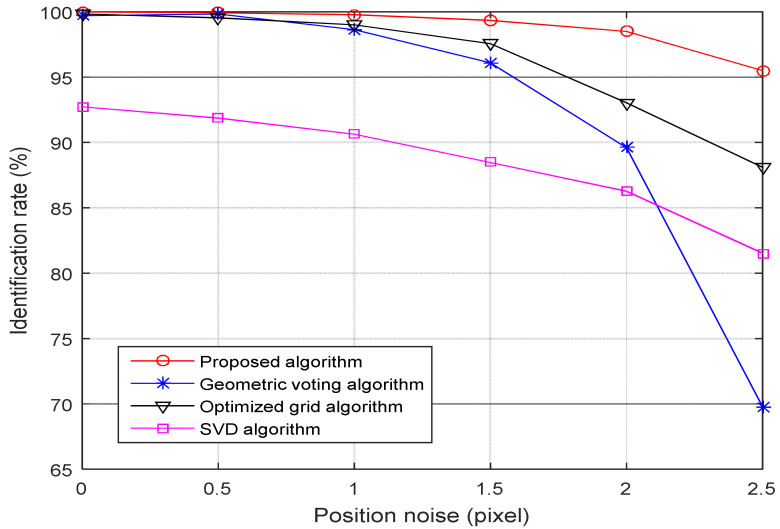
Identification rate versus positional noise.

(1) Robustness toward star positional noise: Figure 6 shows the identification rates for four-star identification algorithms as the standard deviation of the position noise increases from 0.0 to 2.5 pixels, and the standard deviation of the magnitude noise remains at 0.3 Mv.

As shown in Figure 6, the identification rate of the proposed algorithm keeps above 95.50% as the position noise added to the simulated star image increases from 0 to 2.5 pixels. In contrast, the identification rate of the optimized grid algorithm drops slightly, from 99.83% to 88.15%, and the identification rate of the SVD algorithm decreases quickly from 92.72% to 81.56%, while the performance of the geometric voting is poorer, which drops seriously from 99.73% to 69.76%. When the deviation of the position noise is 2.5 pixels, the identification rate of the proposed algorithm is 25.74% higher than the geometric voting algorithm, 7.35% higher than the optimized grid algorithm, and 13.94% higher than the SVD algorithm.

**Figure 7:**
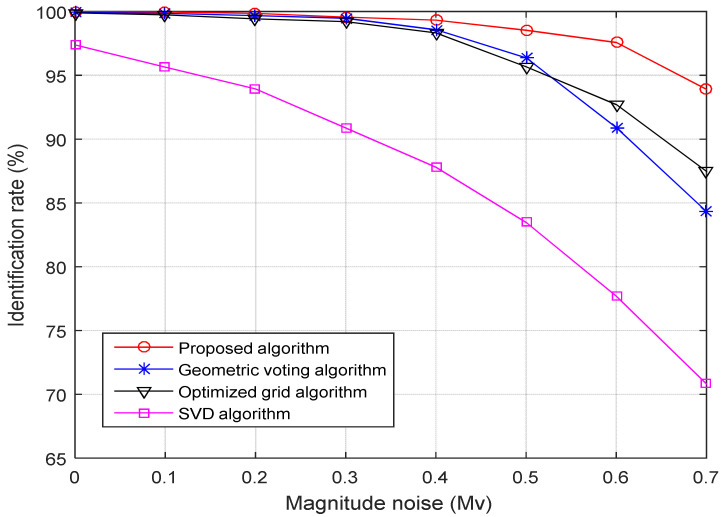
Identification rate versus magnitude noise.

(2) Robustness toward star magnitude noise: Figure 7 shows the identification rates for four-star identification algorithms as the standard deviation of the magnitude noise increases from 0.0 to 0.7 Mv, and the standard deviation of the position noise remains at 1.0 pixels.

As shown in Figure 7, the identification rate of the proposed algorithm drops gradually from 99.98% to 93.88% as the magnitude noise added to the simulated star image increases from 0 to 0.7 Mv. In contrast, the identification rate of both the geometric voting algorithm and the optimized grid algorithm drops quickly, from 99.93% to 84.37% and 99.89% to 87.47%, respectively. The identification rate of the SVD algorithm decreases sharply. It drops from 97.36% to 70.82%. When the deviation of the magnitude noise is 0.7 Mv, the identification rate of the proposed algorithm is 9.51% higher than a geometric voting algorithm, 6.41% higher than the optimized grid algorithm, and 23.06% higher than the SVD algorithm.

**Figure 8:**
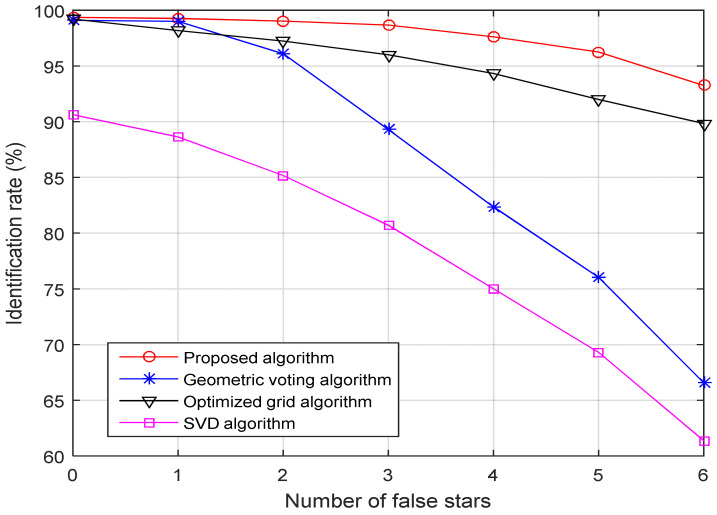
Identification rate versus false stars.

(3) Robustness toward false stars: Figure 8 shows the identification rates for four-star identification algorithms as the number of false stars increases from 0 to 6. The standard deviation of the position noise and magnitude noise is maintained at 1.0 pixels and 0.3 Mv, respectively, and the brightness of false stars varies from 3.5 Mv to 5.5 Mv randomly.

As shown in Figure 8, when the number of false stars is less than 2, the identification rates of the three algorithms are all greater than 95%. As the number of false stars further increases, the identification rate of the geometric voting algorithm and SVD algorithm decreases seriously, much worse than the other two algorithms. For the case of 6 false stars added to the simulated star image, the identification rate of the geometric voting algorithm and SVD algorithm drops to 66.57% and 61.38%, while the identification rate of the optimized grid algorithm and the proposed algorithm is 89.83% and 93.25%, respectively.

(4) Run time and memory size: Table 4 lists the performance results of our method and the other three references in terms of memory cost and runtime. It can be seen that the runtime of our method is 35.3 ms, which is slightly longer than the SVD method (16.2 ms) but largely shorter than the geometric voting method (76.6 ms) and optimized grid method (130.7 ms).

The memory cost refers to the size of the onboard database consisting of a reference catalog and pattern database. It is desired to have a lower database size to reduce memory resources required when the onboard memory is limited in the past decade. However, today’s embedded systems do not have a problem of storing up to 5 MB, and memory cost (less than 5 MB) is not as important as the identification rate and runtime for star identification technique [28]. In fact, the memory size is closely relevant to the number of triangle units constructed by bright stars in FOV. During the database construction, the brightest N stars in FOV are selected to generate $C_{N}^{3}$ triangle units, so we test the effect of increased selected stars number N in FOV on memory size. When N = 6, 8, there are 43,077, 106,303 triangle units in the database and the memory size is 0.5 MB, 1.2 MB, which is less than 1.6 MB and 0.9 MB compared with 2.1 MB memory size under N = 10, respectively. When N = 12, the triangle unit number increases to 396,656, and its memory size is 4.1 MB. Hence, the proposed algorithm requires more storage space than the other three identification algorithms, but it is still acceptable in the application.

(5) Comparison and analysis: Simulation results indicated that the presented method in Section 2 performs better than the three reference methods in terms of the robustness to position noise, magnitude noise and false starts. Besides, its identification speed is also faster than the optimized grid algorithm and geometric voting algorithm.

For the optimized grid algorithm, if the closest neighbor star is wrongly found, the grid pattern will be built in an incorrect orientation, which makes correct matching become almost impossible [7]. In the SVD algorithm, four relative brighter stars from the circle stars are selected. However, some brighter spurious stars or a magnitude error in the actual image may change the original stars in the pattern set and result in identification failure [29]. As to the geometric algorithm, the basic principle of the method lies in the star pairs voting scheme, but the angular distance between stars is low-dimensional information. It is always matched with numerous candidates, which results in the sensor stars easily receive the wrong votes. Moreover, the correct match of a given imaged star pairs may be excluded in its voting process as the measurement error, particularly in the occasion with large position noise. The proposed algorithm is more robust because it adopts the triangle as the unit in the voting process, in which the number of candidates of the voting unit is much less than that of the star pairs. Besides the feature values extracted via SVD transform are stable under position noise, it improves the probability of including correct match in candidates. As a result, the voting scheme utilizing the triangle unit is more anti-noise and reliable, leading to the proposed algorithm can successfully identify the star image where some sensor reference stars are incorrectly selected due to the interference of magnitude error or false stars.

### 3.3. Real Star Image Test

**Figure 9:**
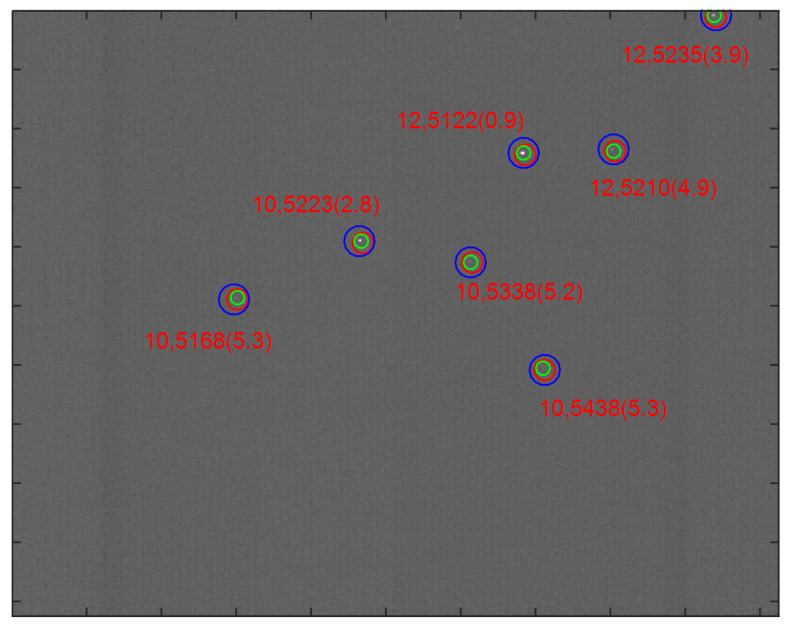
An example of the identification results in real star images.

Besides the simulation test presented in Section 3.2, we also implemented a multilayer voting algorithm on real star images captured by the camera. The camera in the test used an 8 × 8°circular FOV with an image plane consisting of 512 × 512 pixels, and pixel size was 15 μm × 15 μm, and its sensitivity was 5.5 Mv. The star centroiding method was utilized to calculate the coordinates of observed stars in images, and then a multilayer voting algorithm was performed on the 80 real star images of different boresight orientations. The multilayer voting algorithm identified all real star images successfully, and the average run time in an image is 10.68 ms. An example for real star image identification is presented in Figure 9, in which most observed stars are identified by the proposed algorithm, allowing us to determine the attitude of these identified stars.

## 4. Conclusions

This paper presented a multilayer voting algorithm to identify imaged stars in the “lost-in-space” condition for star sensors. In the proposed algorithm, the triangle unit is first utilized as the basic voting unit to obtain the candidates in the initial match, and then a follow-up verification procedure confirms the final matching results. Simulations demonstrate our algorithm obtains a higher identification rate than the optimized grid, geometric voting and SVD algorithms with noise. Moreover, our algorithm is efficient, and the memory size is acceptable. Therefore, the multilayer voting algorithm is applicable for actual star sensors in astronautic missions. We will focus on programming the algorithm into an embedded system to build a real star sensor and test its performance in the future.

## Figures and Tables

**Table 1:** Key parameters in the multilayer voting method and brief descriptions.

Parameter	Description
N	The maximum number of selected stars in a FOV to form the triangle units
$\Delta v_{1}$	The minimum discretized unit in the singular value feature values $v_{1}$
$\Delta v_{2}$	The minimum discretized unit in the singular value feature values $v_{2}$
ver_min	The threshold of the largest cluster group size

**Table 2:** Algorithm functions and brief descriptions.

feature_value (*i*, *j*, *k*)	Calculates the feature values of the triangle unit formed by stars (*i*, *j*, *k*) in the image and returns the feature values ${\hat{v}}_{1}$$,\;{\hat{v}}_{2}$.
search_match_unit ${\hat{v}}_{1}$$,\;{\hat{v}}_{2}$$,\;\mathrm{LT}\_v_{1}$$,\;\mathrm{LT}\_v_{2}$)	According to feature values ${\hat{v}}_{1}$$,\;{\hat{v}}_{2}$ searches the lookup tables $\mathrm{LT}\_v_{1}$, $\mathrm{LT}\_v_{2}$ and finds match units in the catalog, returns the set K, including all match units, and the element number unit_num in K.
unit_starid (K, p)	Determines the three catalog stars indexes in C of the p-th match unit in the K and returns the indexes id1, id2, and id3.
add_counter (t, id1, id2, id3)	The t-th sensor star’s counter of catalog stars that corresponds with id1, id2, and id3 add one and returns the counter of the t-th sensor star.
find_candidate (counter[q])	Determines the candidate of the q-th sensor star with the related counters counter[q] and returns the index of the candidate can[q].
verification (can)	Determines the largest cluster group of the candidates and returns the final identification results.

**Table 3:** An identification example of the algorithm.

Image Star	Catalog Star	Initial Match	Verification
1	147,986	**147,986**	147,986
2	129,825	**129,825**	129,825
3	-	8102	-
4	147,632	**147,632**	147,632
5	110,065	**110,065**	110,065
6	-	163,422	-
7	110,291	**110,291**	110,291
8	148,059	**148,059**	148,059
9	-	258,698	-
10	109,793	13,351	-

The bold number is the correct match for the sensor star.

**Table 4:** Time and Memory Cost.

Algorithm	Our Method	Optimized Grid	SVD	Geometric Voting
Run time	35.3 ms	130.7 ms	16.2 ms	76.6 ms
Memory cost	2.1 MB	0.5 MB	0.15 MB	0.6 MB

## Data Availability

Not applicable.
